# α-Synuclein liquid condensates fuel fibrillar α-synuclein growth

**DOI:** 10.1126/sciadv.adg5663

**Published:** 2023-08-16

**Authors:** Leonard Piroska, Alexis Fenyi, Scott Thomas, Marie-Aude Plamont, Virginie Redeker, Ronald Melki, Zoher Gueroui

**Affiliations:** ^1^ PASTEUR, Department of Chemistry, École Normale Supérieure, PSL University, Sorbonne Université, CNRS, 75005 Paris, France.; ^2^ Institut Francois Jacob (MIRCen), CEA, CNRS, Fontenay-aux-Roses, France.

## Abstract

α-Synuclein (α-Syn) aggregation into fibrils with prion-like features is intimately associated with Lewy pathology and various synucleinopathies. Emerging studies suggest that α-Syn could form liquid condensates through phase separation. The role of these condensates in aggregation and disease remains elusive and the interplay between α-Syn fibrils and α-Syn condensates remains unexplored, possibly due to difficulties in triggering the formation of α-Syn condensates in cells. To address this gap, we developed an assay allowing the controlled assembly/disassembly of α-Syn condensates in cells and studied them upon exposure to preformed α-Syn fibrillar polymorphs. Fibrils triggered the evolution of liquid α-Syn condensates into solid-like structures displaying growing needle-like extensions and exhibiting pathological amyloid hallmarks. No such changes were elicited on α-Syn that did not undergo phase separation. We, therefore, propose a model where α-Syn within condensates fuels exogenous fibrillar seeds growth, thus speeding up the prion-like propagation of pathogenic aggregates.

## Teaser

α-Syn fibrils trigger a liquid-to-solid transition of α-Syn condensates in cells, with needle-shaped amyloid protrusions.

## INTRODUCTION

Biomolecular condensates are subcellular compartments confining proteins and nucleic acids and organizing specific biochemistry in space and time. This particularity entails a high degree of component exchange with the surrounding medium, allowing the modulation of their composition and properties according to different cellular cues (*1*, *2*). Cells make use of these compartments for controlled spatiotemporal colocalization of various factors, whose interactions within the condensates are drivers of fundamental processes such as RNA processing (*3*), cell division (*4*), and response to cellular stress (*5*). While the function and composition are characteristics specific to each condensate or cellular context, their mechanism of formation is increasingly believed to share common principles. For instance, condensate formation is often mediated through multivalent interactions between biomolecules, which could be represented through polymer-based physical models based on phase separation (PS), including liquid-liquid PS (LLPS) (*6*, *7*).

Mounting pieces of evidence tie aberrant behaviors of biocondensates to neurodegenerative diseases (*8*–*11*). It has been hypothesized that LLPS-derived condensates might represent intermediates in the path yielding toxic protein aggregates (*12*, *13*). However, the mechanisms underlying this process associated with aging remain unclear. Rheological experiments performed with purified proteins suggest that in vitro condensates could behave as Maxwell fluids displaying glassy characteristics, implying a time-dependent change in the material properties of condensates (*14*). Alternately, elevated protein concentrations (*15*) and water molecule content reduction within condensates (*16*) were also proposed to drive their aggregation. Reconstitution experiments have revealed that numerous aggregation-prone proteins involved in neuropathological disorders, such as Tau, Fused in Sacroma (FUS), and heterogeneous nuclear RNP A1 (hnRNPA1), phase-separate through LLPS in vitro and in cellulo (*17*–*20*). In addition, in vitro, these proteins underwent a time-dependent progression into solid-like aggregates within condensates (*15*, *17*, *21*). This pattern has sparked the hypothesis that PS is involved in the pathological aggregation of proteins within cells.

In this study, we focused on α-synuclein (α-Syn), a protein whose aggregation is intimately associated with Lewy bodies formation (*22*, *23*) and the synucleinopathies Parkinson’s disease, dementia with Lewy bodies, and multiple system atrophy (*24*–*26*). In vitro α-Syn droplets were generated in the presence of crowding agents or high salt concentration through LLPS and the protein was shown to acquire solid-like properties in a time-dependent manner (*27*–*31*). Besides, some data suggest that α-Syn can form nanoscale structures below the saturation concentration, playing a role in aggregation (*32*). These in vitro observations did not take into account complex biochemical phenomena that take place within cells such as promiscuous interactions, crowding within the cytosol, cellular compartmentalization, and stress (*33*–*35*). One recent study using a *Caenorhabditis elegans* model reported the formation of α-Syn condensates that initially had liquid-like properties and that progressed toward a solid state in aged animals (*36*). Yet, in cellulo work has proven to be difficult, as de novo α-Syn condensates formation could be challenging to monitor using classical optical means. As a consequence, the phase separation of α-Syn in cells and the possible evolution of condensates into pathogenic aggregates remain to be established.

To overcome this obstacle and assess the putative links between α-Syn condensates and their propensity to evolve into aggregates of amyloid nature within cells, we designed a method allowing controlled assembly and disassembly of intracellular liquid condensates enriched in α-Syn and made use of high-resolution microscopy. To drive the formation of phase-separated condensates in cells, we used a self-interacting multivalent protein scaffold based on five consecutive repeats of FKBP12-F36M (hereafter called 5Fm) that we previously showed to undergo PS in cells (*37*). Expression of the fusion protein α-Syn-5Fm in HeLa cells and SH-SY5Y led to α-Syn condensates recapitulating bona fide liquid bodies that could be disassembled upon drug addiction. We next asked how α-Syn condensates evolve in the presence of α-Syn fibrillar assemblies of amyloid nature given that such fibrils have been shown to have prion-like properties (*38*, *39*).

Considering the ability of α-Syn fibrils to amplify through the recruitment of endogenous α-Syn and spread from cell to cell in a prion-like manner (*38*), we characterized the properties of α-Syn within condensates exposed to preformed fibrils. We show here a drastic modification in the morphology of α-Syn condensates upon interaction with preformed fibrils with the acquisition of a “spiky” aspect, reminiscent of sea urchins. We further demonstrate a transition from a liquid-like to a solid-like state. The changes we report were concomitant to the interaction of α-Syn preformed fibrils with the condensates, suggesting seeded liquid-to-solid conversion of α-Syn within the condensates. We establish that α-Syn within converted condensates resists detergents and is phosphorylated, two characteristics of Lewy bodies. We thus postulate that α-Syn condensates could act as a reservoir in the prion-like propagation of α-Syn fibrils.

## RESULTS

### In cellulo formation of α-Syn condensates

**Fig. 1.**
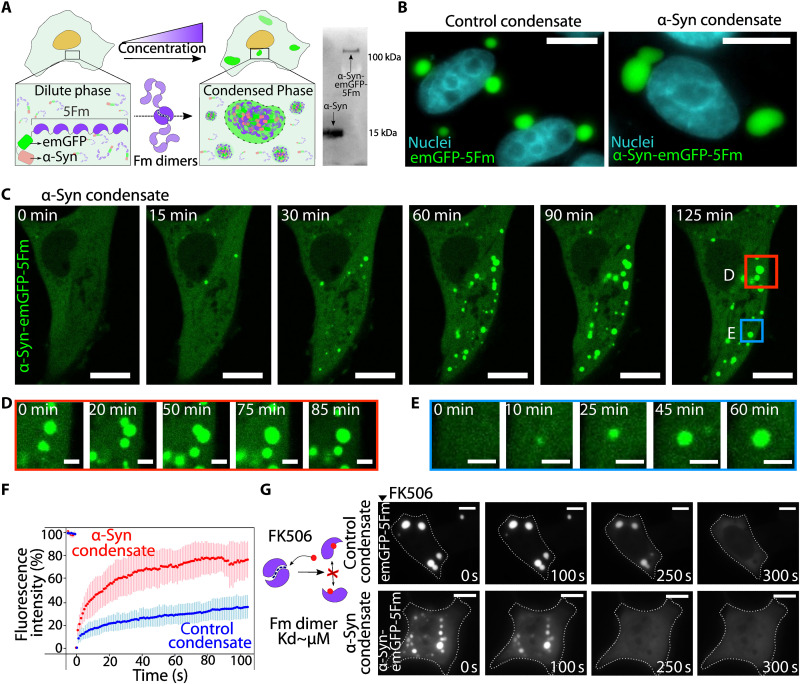
Artificial α-Syn condensates with liquid-like properties. (**A**) Strategy to generate artificial condensates in cells based on a multimeric protein scaffold. Western blot displaying the molecular size of the α-Syn-emGFP-5Fm chimera compared to α-Syn alone. (**B**) Representative epifluorescence imaging of cells expressing control condensates (emGFP5-Fm scaffolds; left, green) and α-Syn condensates (α-Syn-emGFP-5Fm scaffolds, right, green). Scale bars, 10 μm. (**C**) Time-lapse confocal imaging of the formation of α-Syn condensates in HeLa cells starting 10 hours after transfection. Scale bars, 10 μm. (**D** and **E**) Enlarged regions extract from the time lapse (C) displaying growth of condensates mediated by coalescence (D) or α-Syn addition (E). Scale bars, 2 μm. (**F**) Fluorescence recovery after photobleaching performed on α-Syn condensates (red, 12 cells) and control condensates (blue, 17 cells). The dotted line in bold represents the means and the error bar represents the SD. Acquisitions were made every second. (**G**) Time-lapse imaging of the dissolution of 24-hour-old control (upper row) and α-Syn (lower row) condensates upon the addition of FK506. Dashed lines delineate cell boudaries. Scale bars, 10 μm.

To generate α-Syn condensates with modular assembly/disassembly, we fused α-Syn to emerald Green Fluorescent Protein (emGFP)-5Fm, a multivalent protein scaffold designed to form micrometric and spherical condensates when expressed in cells (Fig. 1, A and B, left). Twenty-four hours after transfecting α-Syn-emGFP-5Fm in HeLa cells, we found that α-Syn condensates formed larger micrometric bodies displaying spherical shapes (Fig. 1B, right). To learn more about the α-Syn condensates’ biogenesis, we monitored their formation using time-lapse confocal microscopy. Approximately 10 hours after transfection with the α-Syn-LLPS scaffold, several condensates form throughout the cytosolic space (Fig. 1C and movie S1). As also observed with control condensates (emGFP-5Fm; fig. S1, A to C, and movie S2), α-Syn condensates grew through the coalescence of nearby small condensates forming into larger spherical ones (Fig. 1D) or by addition of scaffolds from the dilute phase into the condensed phases (Fig. 1E). The 5Fm scaffold is required to drive α-Syn condensates formation, as α-Syn-emGFP expression alone yields an even fluorescence distribution (fig. S1D).

We next assessed the dynamic turnover of the α-Syn condensates’ components using fluorescence recovery after photobleaching (FRAP). Typical FRAP curves of the control condensate (emGFP-5Fm) showed a mobile fraction of 40% recovering within 2 min (Fig. 1F). α-Syn condensates exhibited a larger mobile fraction (80%) with an increased dynamical turnover (minute) (Fig. 1F). Moreover, very similar patterns of recovery after photobleaching were observed for α-Syn condensates at 24 and 72 hours after transfection (fig. S1E), suggesting that the presence of α-Syn did not induce a time-dependent change of their dynamic properties within this timeframe. To assess the capacity of α-Syn condensates to disassemble, FK506, a competitive binder that disrupts Fm-Fm dimerization and impairs 5Fm capability to phase separate was added to the cells (*40*, *41*). Live epifluorescence microscopy revealed that 24-hour-old α-Syn condensates dissolved within a few minutes, in the presence of FK506 (Fig. 1G and movie S3). This fast α-Syn condensate dissolution was similarly observed for 72-hour-old α-Syn condensates (fig. S1F and movie S4), suggesting an absence of irreversible assemblies induced by putative strong α-Syn intermolecular interactions within condensates with time. Taken collectively, our data demonstrate our ability to control the assembly/disassembly of liquid α-Syn condensates.

### α-Syn condensates undergo morphological changes in the presence of preformed α-Syn fibrils

**Fig. 2.**
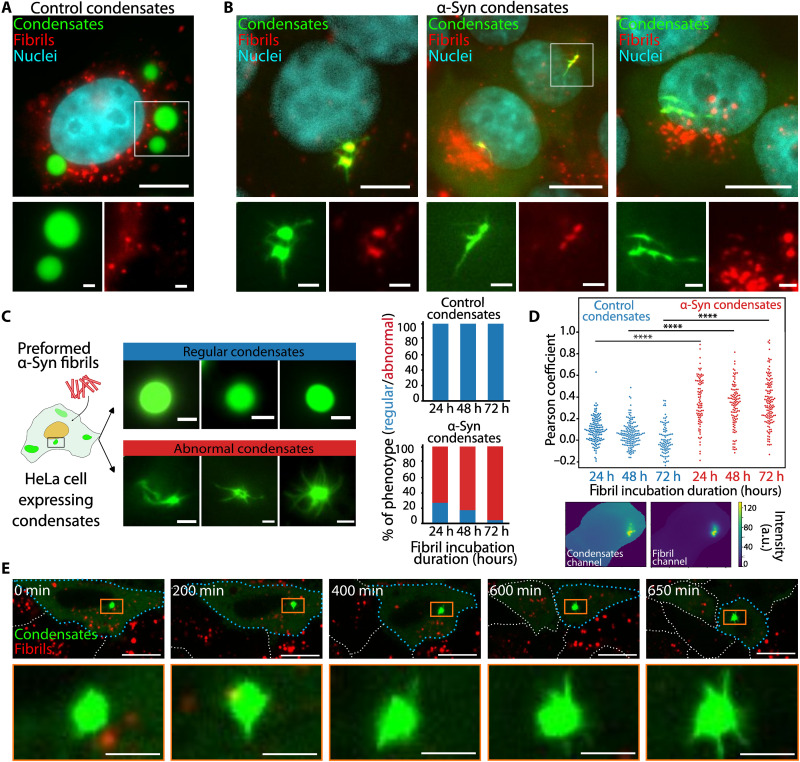
α-Syn condensates are perturbed by preformed fibrils. Representative epifluorescence images of HeLa cells infected with preformed α-Syn fibrils (red) and expressing control (**A**) and α-Syn (**B**) condensates (green). Nuclei (cyan) were stained with Hoechst. Scale bars, 10 μm for full-cell images and 2 μm for zoom-ins. (**C**) Quantification of the percentage of cells with condensates expressing the two different phenotypes: regular, blue; abnormal, red. The graph is the sum of *N* = 3 experiments. (**D**) Quantification of the Pearson’s coefficient calculated for the correlation between the fluorescence intensities of the fibril and condensate channels in microscopy images that were cropped to match the shape of the cell. Each point represents a cell. Differences between conditions with control and α-Syn condensates were statistically significant (*P* < 10^–7^ using a Student’s *t* test). a.u., arbitrary unit. (**E**) Live confocal microscopy frames depicting HeLa cells expressing α-Syn condensates (green) changing morphology upon exposure to fibrils (red). Time 0 corresponds to the time when cells expressing condensates were exposed to fibrils for approximately 48 hours. Dashed lines delineate cell boundaries. Scale bars, 10 μm.

Preformed α-Syn fibrils have been shown to grow by recruiting endogenous α-Syn in cellulo and in vivo. Yet, it remains unknown whether these fibrillar aggregates could affect α-Syn condensates. We therefore set an assay allowing monitoring and quantifying the spatiotemporal dynamics of α-Syn condensates in cells that have been exposed to fluorescently labeled, de novo assembled, α-Syn fibrils (fig. S2). HeLa cells were exposed to fragmented fibrillar α-Syn (0.5 nM) for 24 hours. Observation of those cells by epifluorescence microscopy revealed a notable change in the morphology of the majority of α-Syn condensates within the cells. The condensates displayed nonspherical shape and were often smaller, more anisotropic, with one or multiple bright puncta that appear as nodes for spiky or needle-shaped structures pointing outward (Fig. 2, B and C). No such abnormally shaped condensates were observed in control cells expressing emGFP-5Fm in the presence of fibrils (Fig. 2A). We quantified the incidence of this phenotype as a function of time at 24, 48, and 72 hours. We noticed that α-Syn condensates progressed steadily toward an “abnormal” morphology after fibrils addition, as their proportion rose from 72% at 24 hours to 97% at 72 hours (Fig. 2C). In contrast, cells expressing control condensates (emGFP-5Fm) displayed exclusively spherical condensates irrespective of the exposure time to the same amounts of α-Syn fibrils (Fig. 2C). This suggests that α-Syn within the condensates is responsible of the preformed fibril-mediated morphological alterations that we describe. To determine how preformed α-Syn fibrils trigger a change in condensate morphology, we first assessed whether exogenous α-Syn fibrils and the cellular condensates colocalize. Correlation-based colocalization analyses performed on several hundreds of cells revealed no colocalization between control condensates and exogenous fibrils at any time (Fig. 2D). In contrast, most α-Syn condensates colocalized with exogenous fibrils as early as 24 hours after addition of α-Syn fibrils (Fig. 2D). Furthermore, we found that the efficiency of the fibrils-triggered morphological changes we observed increased with fibrils concentration (0.01, 0.1, and 0.5 nM; fig. S3A). We found that α-Syn preformed oligomers did not trigger morphological changes of α-Syn condensates (fig. S3B), in contrast to preformed α-Syn fibrils. We conclude from these observations that α-Syn condensates somehow remodel in a time- and concentration-dependent manner in the presence of fibrils.

### Abnormally shaped α-Syn condensates originate from fibril-driven maturation of regular condensates

Next, we investigated the time course of α-Syn condensate shape changes by live confocal microscopy in cells containing or not α-Syn fibrils. As indicated above, the micrometric α-Syn-emGFP-5Fm condensate shape evolved with time from spherical to anisotropic, with the formation of multiple needle-like structures sprouting from the condensate core (Fig. 2E and movie S5). This morphological transition occurred within a few hours after the addition of α-Syn fibrils, suggesting that “regular,” spherical, condensates evolve into abnormal, anisotropic ones upon contact with exogenous α-Syn fibrils. Alternately, abnormally shaped condensates may form de novo in the absence of preformed condensates, e.g., upon seeding of diffuse α-Syn-emGFP-5Fm by exogenous α-Syn fibrils (*42*). To determine whether the latter scenario occurs, we exposed cells exhibiting exclusively diffuse α-Syn-emGFP-5Fm to exogenous α-Syn fibrils for 72 hours. No abnormally shaped, anisotropic condensates were observed under these experimental conditions (fig. S3C). Together, our findings suggest that the needle-like extensions we report originate from the recruitment of α-Syn-emGFP-5Fm within preformed condensates, rather than from the seeding of the cytosolic, evenly distributed form of this protein.

### α-Syn fibrils-mediated abnormally shaped condensates have solid-like material properties

**Fig. 3.**
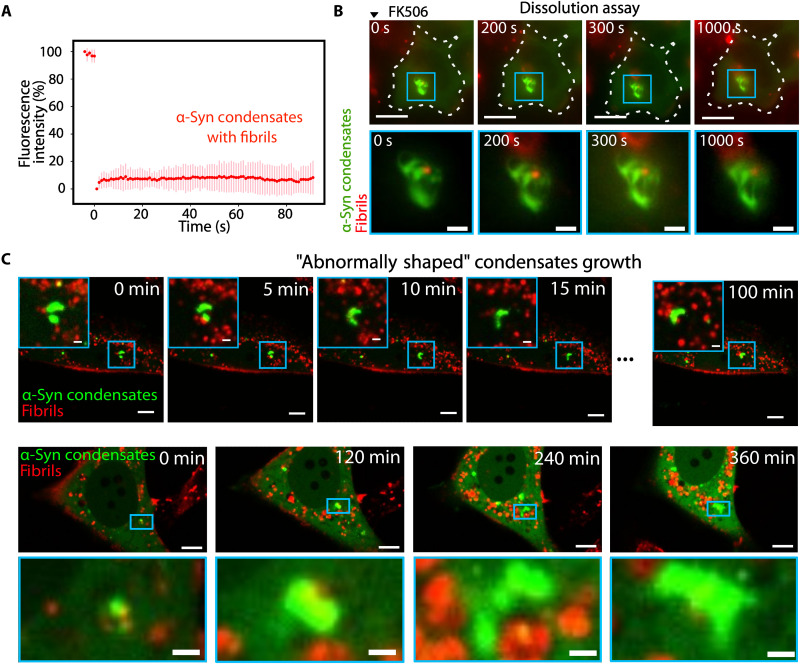
Abnormally shaped α-Syn condensates have solid-like properties. (**A**) FRAP analysis of abnormally shaped condensates. The graph comprises the average of nine different experiments. The prebleaching fluorescence intensity was set at 100 and the postbleaching fluorescence was normalized at 0 for all experiments. An acquisition was made every second. Error bars correspond to the SD. (**B**) Live confocal microscopy frames depicting HeLa cells exposed to fibrils (red) and expressing α-Syn condensates (green) upon exposure to FK506 at a final concentration of 2.5 μM. Different time points are shown, with 0 s being pinpointed at the moment when FK506 was added. Scale bars,10 μm for whole-cell frames where dashed lines delineate cell bounadries and 2 μm for zoom-ins. (**C**) Confocal microscopy live images of the fusion of multiple abnormally shaped condensates (green) without coalescence. Scale bars,10 μm for whole-cell frames and 2 μm for zoom-ins.

To examine potential modifications in the physical characteristics of abnormally shaped condensates, we performed FRAP experiments in cells exposed or not to α-Syn fibrils (24 and 72 hours). Whereas α-Syn condensates in unexposed cells displayed liquid-like properties, photobleached abnormally shaped condensates displayed no recovery of fluorescence (Fig. 3A). The lack of turnover within abnormally shaped condensates evokes a solid-like behavior that was not observed in control spherical condensates from cells exposed or not to α-Syn fibrils (fig. S3D). We conclude from these observations that α-Syn condensates interaction with α-Syn fibrils triggers a liquid-to-solid–like transition.

Next, we assessed the dissolution of α-Syn condensates in the presence of FK506. Abnormally shaped α-Syn condensates resisted prolonged FK506 treatment (over 1 hour) (Fig. 3B, fig. S3E, and movie S6) in sharp contrast with regular α-Syn condensates within cells unexposed to α-Syn fibrils (Fig. 1G). The resistance to dissolution was correlated with the morphological state of the condensates, as the subset of regular α-Syn condensates underwent dissolution in contrast with abnormally shaped ones within the same cells (fig. S3F). In a few cases, some condensates were found to be heterogeneous at the micrometer scale, with small abnormally shaped assemblies engulfed into a larger spherical α-Syn condensate. Consistent with the shape-reversibility relationship, we observed FK506-mediated dissolution of the spherical, not the abnormally shaped condensates (fig. S3F). An additional characteristic of the α-Syn fibril–mediated changes in condensates’ properties was seen upon the fusion of two close abnormally shaped α-Syn condensates. These condensates did not yield a spherical body upon coalescence, as generally seen for liquid condensates, even after prolonged contact (hours) (Fig. 3C and movie S7*).* The lack of relaxation suggests an absence of internal rearrangements at the micrometer scale (Fig. 3C). Together, our findings suggest that strong bonds within condensates are sufficient to counterbalance relaxation forces and stabilize abnormally shaped condensates against micrometer-scale reorganization and dissolution.

### Abnormally shaped α-Syn condensates exhibit amyloid-like properties

**Fig. 4.**
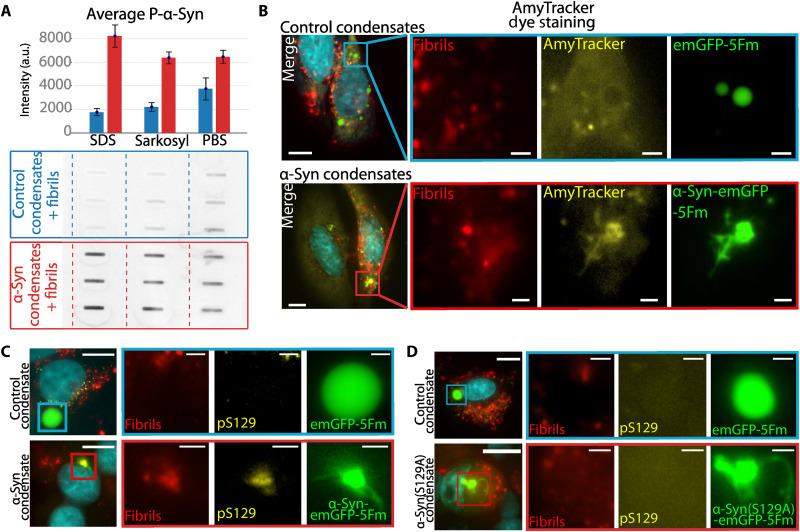
Abnormally shaped α-Syn condensates are of amyloid nature. (**A**) Filter trap analysis coupled with immunoblotting against α-Syn-pS129 performed on cell lysates containing condensates incubated with preformed fibrils for 72 hours. The bar plot illustrates the chemiluminescence intensity for immunoblotting. Results from control condensates are in blue and results from α-Syn condensates are in red. For each of the three experimental conditions [SDS, sarkosyl, and phosphate-buffered saline (PBS)], *N* = 3 experiments were performed with control and α-Syn condensates. Error bars represent the SD. (**B**) AmyTracker staining on HeLa cells expressing α-Syn condensates and control condensates after 72-hour incubation with fibrils. (**C**) Epifluorescence microscopy images depicting HeLa cells exposed to wild-type α-Syn fibrils and expressing condensates after immunostaining for α-Syn-pS129. Nuclei were stained with Hoechst. (**D**) Epifluorescence microscopy images depicting HeLa cells infected with α-Syn(S129A) fibrils and expressing α-Syn(S129A) condensates after immunostaining for α-Syn-pS129. Nuclei were stained with Hoechst. Scale bars, 10 μm for whole-cell images and 2 μm for zoom-ins.

**Fig. 5.**
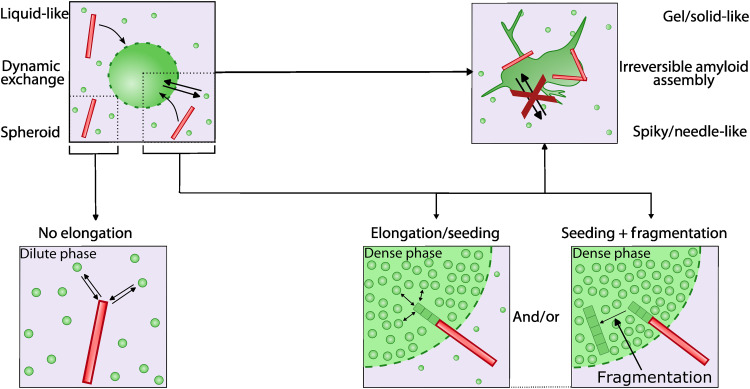
Illustration of the hypothesized mechanisms underlying the liquid-to-solid transition of α-Syn condensates in the presence of fibrils. Schematic of a single α-Syn condensate (green droplet) in dynamical equilibrium with its dilute phase. Green dots are monomeric α-Syn-emGFP-5Fm exchanging between the cytosol and the condensate. The elongation of exogenous fibrillar α-Syn polymorphs (red rods) is not as efficient in an environment containing physiological concentrations of α-Syn (bottom, left). In contrast, the elongation of exogenous fibrils is explosive in the highly concentrated environment of liquid condensates (bottom, middle and right). Preformed fibrils (red) elongate rapidly by incorporation of α-Syn-emGFP-5Fm (green). Explosive elongation may lead to imperfect stacking and breakage of long fibrils into shorter ones. Other factors, known to fragment fibrils such as molecular chaperones, trapped within the condensates, may sever the fibrils leading to an increased number of fibril ends and aggregation. Last, changes in the viscoelastic properties of the condensate environment may favor the de novo aggregation of α-Syn-emGFP-5Fm. Together, these events drive a liquid-to-solid transition and the resulting changes in condensate shape.

To further assess the biochemical properties of the abnormally shaped α-Syn condensates, we first ensured that the protein profiles and concentrations from three independent cell cultures expressing α-Syn-emGFP-5Fm or emGFP-5Fm exposed to exogenous fibrils were similar. To this end, SDS–polyacrylamide gel electrophoresis (PAGE) was performed (fig. S4A). We next demonstrated by Western blot analysis and using an anti-pS129 α-Syn antibody that α-Syn-emGFP-5Fm is phosphorylated in abnormally shaped condensates, not in control liquid counterparts (figs. S4, B and D). We also found that the quantities of preformed fibrils in cells were negligible in regard to the expressed α-Syn-emGFP-5Fm constructs (fig. S4C). We further showed using a filter retardation assay that α-Syn-emGFP-5Fm acquired resistance to detergent, upon cell exposure for 72 hours to exogenous fibrils, not in their absence (Fig. 4A). This suggests that they are of amyloid nature (Fig. 5). No such detergent-resistant aggregates were detected upon exposure of cells expressing emGFP-5Fm control condensates to exogenous α-Syn fibrils. The amyloid nature and/or content of the abnormally shaped α-Syn-emGFP-5Fm condensates was further confirmed by staining with AmyTracker (Fig. 4B). The phosphorylation of α-Syn-emGFP-5Fm was confirmed in cellulo by immunofluorescence (Fig. 4C). Collectively, these observations indicate that α-Syn-emGFP-5Fm condensates are remodeled microscopically upon interaction with exogenous α-Syn fibrils. This process is accompanied by the acquisition of biochemical properties that recapitulate the phenotypic markers of pathological α-Syn inclusions, including S129 phosphorylation and detergent resistance.

### Liquid-to-solid transition is independent from α-Syn phosphorylation within condensates

To determine whether the phosphorylation of residue S129 within α-Syn is required for liquid-to-solid transition within α-Syn condensates, we generated α-Syn condensates where serine-129 was changed to alanine which prevented phosphorylation. Classical spherical condensates were observed in cells expressing α-SynS129A-emGFP-5Fm. Those condensates displayed drastic morphological changes, phenocopying those found in cells expressing wild-type (WT) α-Syn condensates upon cell exposure to exogenous S129A α-Syn fibrils (Fig. 4D, bottom). This implies that the liquid-to-solid transition of α-Syn condensates is independent of the phosphorylation of α-Syn within condensates. Furthermore, the finding that exogenous fibrils, made of α-Syn that cannot be phosphorylated, are equally potent in inducing abnormally shaped condensates within cells shows that exogenous fibril phosphorylation, which we showed not to occur, is not responsible for α-Syn condensates remodeling (*43*, *44*).

### Fibrillar α-Syn polymorphism and the formation of abnormally shaped condensates

α-Syn assembles into distinct fibrillar polymorphs that bind neuronal cells and seed the aggregation of endogenous α-Syn differently (*42*). To determine whether structurally distinct α-Syn fibrillar assemblies remodel α-Syn-emGFP-5Fm condensates, we exposed cells expressing α-Syn condensates to the α-Syn fibrillar polymorph ribbons. This leads to morphological alterations within α-Syn condensates, which were not observed with control condensates, similar to those triggered by the polymorph fibrils (fig. S5 and movie S8). We conclude from this observation that structurally distinct α-Syn fibrillar polymorphs are capable of remodeling α-Syn-emGFP-5Fm condensates in a time-dependent manner (fig. S5E), most probably through a seeding process.

### Preformed α-Syn fibrils triggered the evolution of liquid α-Syn condensates into solid-needle like structures in SH-SY5Y neuronal cells

To determine whether the conversion of liquid α-Syn condensates into abnormal ones may occur in other cell lines, we transposed our assay into the neuronal cells SH-SY5Y. We found that α-Syn-emGFP-5Fm expression in SH-SY5Y cells led to the formation of very large condensates dispersed throughout their cytoplasm (fig. S6A). We observed the evolution of liquid α-Syn condensates into needle-like structures in SH-SY5Y cells exposed either to fibrils or ribbons, thus phenocopying our observations in HeLa cells (fig. S6B). Abnormal condensates were also positive to the AmyTracker stain (fig. S6B) and FRAP experiments indicated that they have solid-like properties (fig. S6C).

## DISCUSSION

In recent years, several proteins involved in neurodegenerative diseases, such as Tau, TDP43, FUS, huntingtin, and hnRNPA1, have been reported to form cytosolic condensates, and efforts have been made to describe the physiological and pathological consequences of this phenomenon. Little evidence supporting α-Syn PS in a cellular context has been brought so far. Nonetheless, α-Syn is predicted to undergo PS given (i) its overall low complexity and the specific abundance of charged and polar amino acid residues within its C-terminal domain (*24*, *45*, *46*), (ii) its ability to form condensates at high concentrations and in the presence of crowding factors in vitro (*27*, *28*) which have been proposed to favor fibrils formation (*27*, *47*), and (iii) its presence within Tau and synapsin condensates (*48*, *49*). Studies in cellular models have proven to be difficult, with limited reproducibility, thus motivating our design of artificial α-Syn condensates. Biophysical characterizations indicated that our artificial α-Syn condensates recapitulated the spheroid morphology, the reversibility, and the liquid-like material properties that characterize PS-derived cellular compartments. This confirmed that our artificial condensates could be instrumentalized to decipher the impact of PS on the protein.

For proteins such as FUS or Tau, phase-separated droplet formation in vitro is sufficient to trigger the spontaneous nucleation and subsequent growth of fibrillar aggregates (*15*, *20*). This temporal evolution from liquid states to more gel or solid ones has fueled the hypothesis that liquid condensates act as intermediates between diffusible monomers and stable, disease-associated aggregates by providing an aggregation-suitable environment, notably through concentrating aggregation-prone proteins (*15*, *20*). Recent in vitro studies and theoretical modeling examined how liquid condensates could modulate nucleation rate. For instance, coacervate droplets modified α-Syn aggregation properties by accelerating their formation at their surface or, in contrast, by suppressing aggregation within droplets (*50*). The interface of droplets was also shown to accelerate fibril formation of the low-complexity domain of hnRNPA1 (*46*). Studies of α-Syn droplets coupled to a thioflavin T assay suggest that aggregation within droplets could appear from primary nucleation and that secondary processes due to changes in viscosity, concentration, hydration, interface, etc. could outperform primary ones (*28*). Furthermore, theoretical predictions also showed how spatially localized aggregation within liquid condensates could emerge from weak interactions between protein monomers and the compartments that could act as continuous sinks (*47*).

In our setting, α-Syn artificial condensates behaved as highly dynamic bodies displaying no time-dependent evolution in material properties over 72 hours. These persistent liquid-like properties suggest no detectable protein aggregation events within our condensates over this time scale. Two previous studies found that α-Syn condensates could undergo maturation over time. In HeLa cells, α-Syn condensates behave as liquid-like bodies at 24 hours, with a decrease in the dynamic at 48 hours (*27*). In the nematode *C. elegans*, α-Syn condensates display liquid-like properties in young worms (up to 7 to 11 days) and evolved toward gel inclusions in aged worms (15 days) (*36*). Differences in behavior with our α-Syn artificial condensates may be due to an antiaggregation propensity of the emGFP-5Fm moieties grafted to α-Syn protein within the condensates. Together, this agrees with the idea that the initial nucleation events of fibril aggregates assembly can be viewed as a rate-limiting step for the assembly process (*51*).

Primary nucleation and subsequent growth of aggregates are not, however, the only pathways for synucleinopathy expansion. Assessment of α-Syn pathology progression in the brain using preformed α-Syn fibrils has revealed that fibrillar α-Syn can be excreted from affected cells into the extracellular environment and taken up by naïve cells (*38*). Pieces of evidence for the active transport of fibrillar α-Syn aggregates through membranous structures between neuronal cells have also been brought (*52*, *53*). Fibrillar α-Syn multiplies by recruiting their cellular monomeric counterpart while spreading, thus contributing to disease progression via this prion-like propagation process (*39*). It remains nonetheless unclear whether fibrils could interact with α-Syn within condensates in cells and what potential outcomes could emerge from such interplay. Thanks to our assay, one could probe how fibrillar forms of α-Syn affect a monomeric α-Syn inside condensates within cells. We found that the interactions of fibrils with the condensates trigger severe changes in their morphology, which entailed the formation of micrometer-long needle-like protrusions originating from α-Syn condensates. These modifications were accompanied by a conversion of α-Syn from a dynamic liquid state to a solid-like state exhibiting the characteristics of amyloid deposits, namely, irreversibility, arrested component turnover, and resistance to SDS.

These abnormally shaped bodies originated exclusively from already-formed condensates, indicating that α-Syn within PS-derived condensates is more prone than its diffuse cytosolic counterpart to undergo conversion into amyloid fibrils. In addition, the abnormally shaped condensates stained positive for α-Syn phosphorylated at serine-129, a hallmark of pathogenic α-Syn within Lewy bodies (*23*, *54*, *55*). This further supports the view that α-Syn within the condensates can fuel α-Syn fibrils growth and multiplication and the spreading of pathology within the central nervous system. A critical aspect in α-synucleinopathies is the concept of strains where different α-Syn fibrillar polymorphs impose their intrinsic structural characteristics upon recruitment of endogenous α-Syn (*42*). As a perspective, it will be interesting to examine whether the needle-shaped amyloid structures that form in cells from the condensates retain the structural properties of the seed.

What makes α-Syn condensates more prone than the diffusing α-Syn pool to aggregate upon fibril exposure? We propose that supersaturation is achieved by α-Syn condensation (Fig. 5) (*56*, *57*). Preformed fibrils can elongate by recruiting monomeric α-Syn. Seeding and preformed fibril growth are notably less efficient in a dilute environment containing physiological α-Syn as compared to that of the highly concentrated liquid α-Syn condensates (Fig. 5). This accounts most for our observations. Furthermore, fragmentations of rapidly growing fibrils may generate additional ends, leading to further acceleration of liquid-to-solid transition and the resulting changes in α-Syn condensate shape. This phenomenon could be due to imperfect stacking of α-Syn in the fibrils, severing factors such as molecular chaperones within the condensates, and/or de novo aggregation of α-Syn-emGFP-5Fm because of changes in the viscoelastic properties of the condensate environment (Fig. 5). Although supersaturation could also lead to de novo aggregation without exogenous α-Syn fibrillar triggers, this was not observed with α-Syn condensates in the absence of preformed fibrils. This may be due to the antiaggregation propensity of the emGFP-5Fm moieties of α-Syn-emGFP-5Fm molecules within the condensates. This suggests that our α-Syn condensates are at a concentration that is high enough to substantially accelerate exogenous fibril-induced aggregation, but that does not exceed the threshold for spontaneous aggregation (*56*). It is further reasonable to hypothesize that the extent to which α-Syn conformational states compatible with fibrillar assembly formation or the elongation of preformed fibrils are populated is much higher within α-Syn condensates than in the diffusing α-Syn pool (Fig. 5). Both pictures could account for the drastic morphological changes and time-dependent evolutions of α-Syn-emGFP-5Fm condensates enabling the liquid-to-solid transition (Fig. 5).

How our observations could be related to the aggregation of α-Syn in neurons? Our artificial α-Syn condensates form micrometric bodies in HeLa and SH-SY5Y cells that are unlikely to be of similar sizes within neurons. Yet, phase-separated condensates have recently attracted strong interest in the physiology and pathophysiology of neurons (*58*). For instance, in vitro studies suggested that synaptic proteins, such as postsynaptic density or synapsin, could phase-separate, thus proposing a complementary framework to examine assemblies at synapses (*48*). In this context, it has been shown that overexpression of synapsin can trigger condensation and recruit α-Syn by partitioning, which could, in turn, potentially modulate synapsin/synaptic vesicle condensation and clustering (*48*). Therefore, the perturbation of synaptic activity described during the accumulation of insoluble α-Syn might be mediated by such condensates. In addition, it has been hypothesized that the irreversible evolution of α-Syn condensates in the presence of lipids and other cellular components in the *C. elegans* model might be linked to the formation of Lewy bodies, one of the main landmarks of Parkinson’s disease (*36*). Last, the up-regulation of *SNCA* gene expression in human induced pluripotent stem cell–derived dopaminergic neurons was observed upon seeded aggregation of endogenous α-Syn (*43*) and may favor α-Syn phase transition.

Our findings show how α-Syn–enriched condensates could favor the prion-like propagation of pathogenic α-Syn fibrillar aggregates. Our ability to fine-tune seeding within our assay together with its sensitivity and robustness make it amenable to high-throughput screening of modulators of α-Syn aggregation. Furthermore, the flexibility of our system could allow the investigation of other neurodegeneration-prone proteins to address the fundamental hypothesis linking liquid condensates and aggregation.

## MATERIALS AND METHODS

### Cell culture

All cellular experiments were carried out in human epithelioid carcinoma HeLa (American Type Culture Collection, ccl-2) cells and SH-SY5Y(ECACC). Cells were maintained in Dulbecco’s modified Eagle’s medium [DMEM; with d-glucose (4.5 g/liter); Corning, 10-017-CV] supplemented with 10% fetal bovine serum (FBS; Gibco, 10270-106) and 1% penicillin/streptavidin (P/S; Sigma-Aldrich, P4333), at 37°C in a 5% CO_2_-humidified incubator. Cells were tested every 2 months for mycoplasma infection. SH-SY5Y was a gift from I. Janoueix-Lesosey (Institut Curie).

### Plasmids

The pcDNA3.1 (Invitrogen) backbone was used for all the plasmids transfected during this study (table S1). All plasmids contain the 6His and the Myc tag downstream of the coding sequence. The pcDNA3.1-emGFP-5Fm plasmid was created by inserting the emGFP coding sequence between the HindIII and Eco47III restriction sites from the pcDNA3.1-5Fm. The pcDNA3.1-α-Syn-emGFP-5Fm plasmid was created by replacing the seipin coding sequence from the pcDNA3.1-Seipin-emGFP-5Fm (between the HindIII and SacII restriction sites) with the α-Syn coding sequence. The pcDNA3.1-α-Syn(S129A)-emGFP-5Fm plasmid was created from the pcDNA3.1-α-SynWT-emGFP-5Fm using a primer containing the S129A mutation through the Gibson method.

### Transfection

All transfection experiments were carried out using Lipofectamine 2000 (Invitrogen) and OptiMEM (Gibco, 31985-062). Cells were seeded for 24 hours before transfection (80,000 cells per well on 12 mm diameter round coverslips for 24-well plates; 350,000 cells per well on 22 × 22 mm^2^ coverslips for six-well plates and 100,000 cells per well for ibidi dishes) and placed in the incubator at 37°C and 5% CO_2_. For each well, two solutions were mixed together: Solution 1 contained Lipofectamine 2000 (2 μl for 24 wells or ibidi dishes and 4 μl for 6 wells) + optiMEM (50 μl for 24 wells, 60 μl for ibidi dishes, and 150 μl for 6 wells); solution 2 contained plasmid DNA (750 ng for 24 wells, 1 μg for ibidi dishes, and 2 μg for 6 wells) + OptiMEM (same volumes as solution 1). Solution 1 was incubated for 5 min before mixing with solution 2 and incubating for another 20 min. The cells were then incubated with DNA/Lipofectamine 2000 mix for 1 hour and 30 min at 37°C and 5% CO_2_, then washed twice with DMEM + Fetal Bovine Serum (FBS) + P/S and incubated for 24 to 72 hours depending on the experiment. Each of the three plasmids, i.e., pcDNA3.1-emGFP-5Fm, α-Syn-emGFP-5Fm, and α-Syn(S129A)-emGFP-5Fm, were cotransfected with the 5Fm in 1:1 stoichiometry, except for experiments carried out in six-well plates where the control emGFP-5Fm was cotransfected with 5Fm in a 2:3 stoichiometry to avoid the saturation of the images.

### α-Syn seeding assays

Human WT α-Syn and a version of the protein where the phosphorylatable Ser^129^ residue was replaced by an Ala were expressed, purified, and assembled into the fibrillar polymorphs fibrils and ribbons as described previously (*42*). The fibrillar polymorphs were diluted to 0.5 nM in DMEM + SVF + P/S and added to cells for 1 hour and 30 min after transfection (at the moment when the Lipofectamine 2000 + DNA mixture was washed away).

For fixed cells experiments, 24-well plates were used. Cells were incubated for 24 hours with fibrils at 37°C and 5% CO_2_ for fibril integration, then washed with fresh DMEM + FBS + P/S. They were then either fixed immediately after washing (for 24-hour fibril incubation experiments) or incubated for another 24 to 48 hours (for 48- and 72-hour fibril incubation experiments, respectively) before fixation and observation on an epifluorescence microscope. For details about the experimental setting, please refer to the “Imaging” section.

For live-microscopy experiments, ibidi μ-Dish (35 mm) was used. Cells with fibrils were followed through overnight movies at different time points: For the 0- to 24-hour interval, cells were incubated for 7 to 8 hours with fibrils, and then transferred to a confocal microscope. For 24 to 48 hours, cells were incubated for 24 hours with fibrils, washed with fresh medium, and then transferred to the confocal microscope. For 48- to 72-hour experiments, cells were incubated with fibrils for 24 hours, washed, then incubated for another 24 hours before another round of wash and the transfer to the confocal microscope. For details about the experimental setting, please refer to the “Imaging” section.

### Immunofluorescence

Twenty-four to 72 hours after transfection, cells were fixed with a 4% paraformaldehyde solution (Sigma-Aldrich) for 20 min. Cells were then washed three times with phosphate-buffered saline (PBS) and permeabilized with a solution of 0.1% Triton X-100 (Sigma-Aldrich) for 10 min, washed three times for 5 min with PBS, and then incubated for 1 hour with the primary antibody anti–α-Syn-pS129 (Abcam, ab51253) diluted 1:10,000 in a solution of 1% BSA in PBS. Then, cells were washed again three times for 5 min with PBS and incubated with the secondary antibody Alexa Fluor 568 (Invitrogen, A-11011) diluted 1:1000 in a solution of 1% BSA in PBS. Cells were then washed again three times for 5 min in Dulbecco’s PBS and mounted on coverslips using the Fluoromount-G mounting medium.

### Dissolution and reversibility and granule formation prevention assays

For live dissolution, a 2.5 mM solution of FK506 (in dimethyl sulfoxide) was diluted in 50 μl of medium and was added to the cells such that the final concentration was 2.5 μM. Dissolution was monitored by capturing an image every 10 s starting from FK506 addition. For condensate formation prevention, FK506 at a final concentration of 2.5 μM was added to cell media 1 hour and 30 min after transfection (at washing). For condensate reversibility assays, FK506 at a final concentration of 2.5 μM was added to cell media 1 hour before fixation.

### Fluorescence recovery after photobleaching

FRAP experiments were carried out in live HeLa cells in a humidified chamber maintained at 37°C and 5% CO_2_, which was mounted on a confocal microscope (see details in the “Imaging” section). Condensates were scanned 10 times to establish the average level of initial fluorescence, then bleached using a 488 nm laser at 100% intensity (10 iterations). The recovery of fluorescence was then followed by acquiring an image of 512 × 512 pixels every second for 120 s. The FRAP movies were analyzed using the Fiji software where each condensate studied was integrated into a region of interest from which the average fluorescence intensity was extracted. To correct the movement of the condensates, the StackReg plugin was used on some of the movies. Analysis and fitting of the fluorescence intensity data were performed using Python. For the graphical representation, we fixed the initial fluorescence at 100% and the fluorescence at bleaching 0% and normalized the remaining points accordingly.

### Western blot analysis

For Western blots, cellular pellets were resuspended in PBS buffer supplemented with 0.5% Tween 20 and lysed using a Branson Sonifier (approximately five to seven pulses of 15 s at 60% amplitude). Cell homogenates were denatured with preheated (95°C) sample buffer [50 mM tris-HCl (pH 6.8), 4% SDS, 2% ß-mercaptoethanol, 12% glycerol, and 0.01% bromophenol blue]. After a 5 min incubation at 95°C, the samples were analyzed on 12% SDS-PAGE, followed by a transfer to a polyvinylidene difluoride membrane for 2 hours at 30 V. The membranes were blocked in 5% skimmed milk and probed with the primary antibody overnight and with the secondary antibody for 1 hour (both at 4°C). Chemiluminescence was imaged and quantified using the Clarity Western ECL Substrate (Bio-Rad) in a ChemiDoc Imaging System (Bio-Rad).

### Quantification of pathogenic α-Syn using Western blot analysis and filter-retention assay

Pathogenic α-Syn was quantified using both a filter retardation assay and immunoblotting and Western blot analysis. Cell homogenates in PBS, PBS containing 1% sarkosyl, and PBS containing 1% SDS (0.5 mg) were immobilized on cellulose acetate membranes (0.2 μm pore size; Millipore Corp., Bedford, MA) by filtration using a 48-slot slot-blot filtration apparatus (GE Healthcare). The membranes were blocked in 5% skimmed milk and probed with the anti-pS129 α-Syn antibody EP1536Y (Abcam, catalog no. ab51253) and a goat anti-rabbit antibody coupled to HRP (Thermo Fisher Scientific, catalog no. A27036).

### Imaging

Fixed samples and dissolution movies were imaged with an epifluorescence microscope (IX81, Olympus) operated by the Micro-Manager 2.0, using an Orca-fusion camera (Hamamatsu), a 63× oil-immersion objective, and the Spectra-X Lumencor LED for illumination. For overnight live movies and FRAP, we used the Zeiss LSM 710 META laser scanning confocal microscope equipped with a humidified chamber maintained at 37°C and 5% CO_2_. Samples were illuminated using a 25 mW argon laser and observed using a 63× oil-immersion objective. The microscope was operated using the LSM Zen 2012 software.

### Statistical analysis

For Fig. 2D and fig. S5D, a Python script based on the scipy.stats.pearsonr function was used to determine the Pearson’s coefficient describing the linear relationship between the fluorescence intensity within the fibril and condensate channels. To assess the statistical significance of the data, Student’s *t* tests were performed for control and α-Syn condensates data samples from the same time point. All datasets were confirmed to be normal distributions before analysis with Student’s *t* test.
