# Supplementary material for: α-Synuclein liquid condensates fuel fibrillar α-synuclein growth
Source: Sci Adv. 2023 Aug 16;9(33):eadg5663. doi: 10.1126/sciadv.adg5663 (PMC10431715; doi:10.1126/sciadv.adg5663)
Supplement: Supplementary file 1 — Figs. S1 to S6 Table S1 Legends for movies S1 to S8 [file sciadv.adg5663_sm.pdf]

Science Advances
AAAS

Supplementary Materials for

α-Synuclein liquid condensates fuel fibrillar α-synuclein growth

Leonard Piroska *et al.*

Corresponding author: Ronald Melki, ronald.melki@cnrs.fr; Zoher Gueroui, zoher.gueroui@ens.fr

Sci. Adv. **9**, eadg5663 (2023)
DOI: 10.1126/sciadv.adg5663

The PDF file includes:

Figs. S1 to S6
Table S1
Legends for movies S1 to S8

Other Supplementary Material for this manuscript includes the following:

Movies S1 to S8

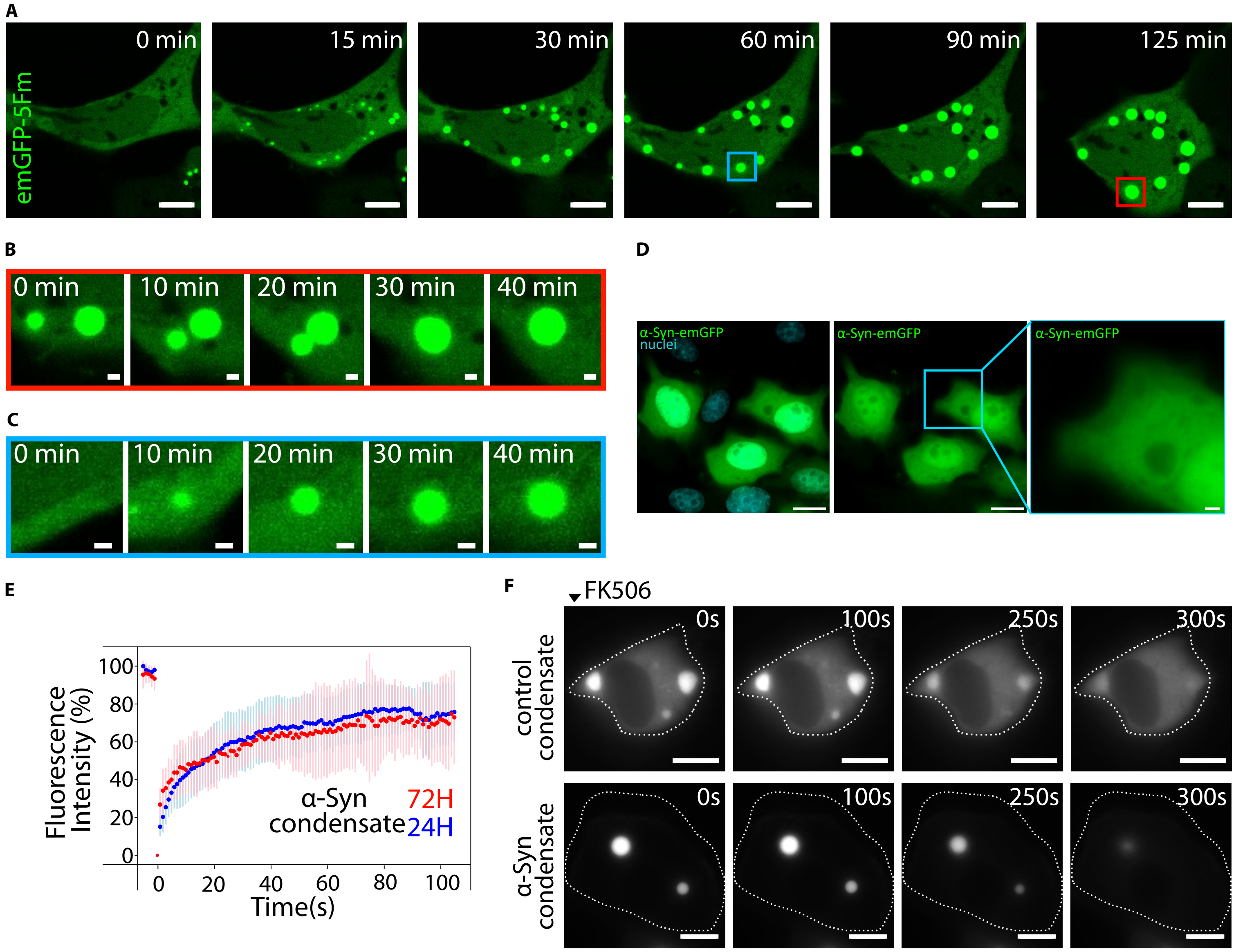

Fig. S1. Artificial α-Syn and control condensates with liquid-like properties

(**A**) Time-lapse confocal imaging of the formation of control condensates in HeLa cells starting from 10h after transfection. Bar, 10µm. (**B**) and (**C**) Enlarged regions extract from the timelapse (**A**) displaying growth of condensates mediated by coalescence (**B**) or subunit addition (**C**). Bars, 2µm. (**D**) Epi-fluorescence microscopy images of HeLa cells expessing the α-Syn-emGFP fusion. Bar, 10µm for whole cell images and 2µm for zoom-ins. (**E**) FRAP curves displaying the fluorescence recovery of α-Syn-enriched condensates 24H (blue, 10 cells) and 72H (red, 15 cells) after cell transfection with the α-Syn_emGFP_5Fm scaffold. Dotted line in bold represents the mean and the error bar represents the standard deviation. Aquisitions were made every second. Pre-bleaching fluorescence intensity was set at 100 and the post-bleach fluorescence was normalized at 0 for all experiments. (**F**) Time-lapse imaging of the dissolution of 72H-old control (Upper row) and α-Syn (Lower row) condensates upon the addition of FK506. Bar, 10µm.

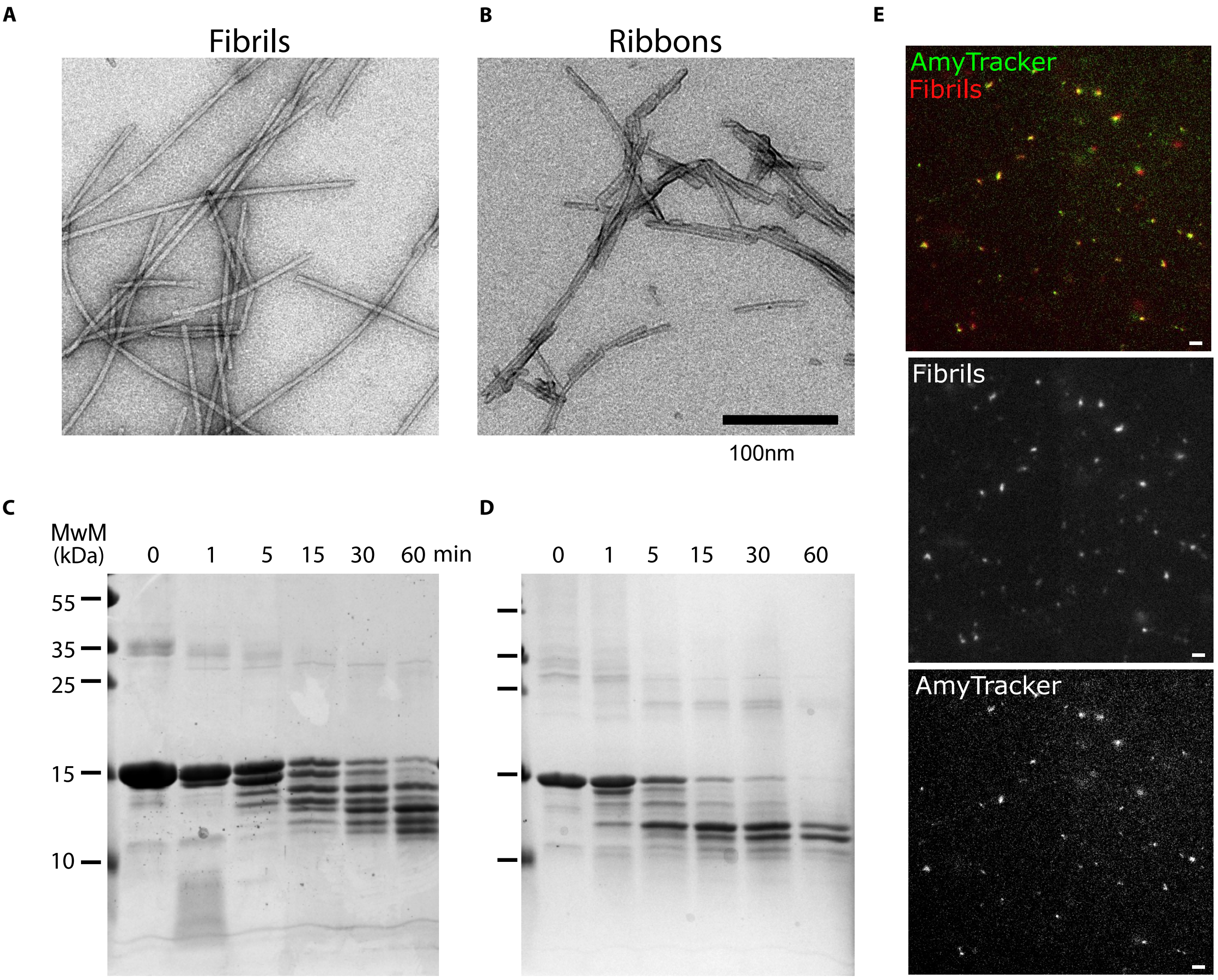

Fig. S2 Characterization of the α-syn fibrillar polymorphs.
Fibrils and Ribbons used throughout this study. Transmission electron micrographs of the polymorphs Fibrils and Ribbons after negative staining with uranyl acetate (panels **A** and **B**). Time course of proteolysis of the polymorphs Fibrils and Ribbons by proteinase K (3.8 µg/mL) after PAGE and staining with Coomassie blue (panels **C** and **D**). Bar, 100nm. The time is in min and the molecular weight markers, in kDa, are indicated on the top and left of each polyacrylamide gel. (**E**) Observation of preformed fibrils (0.5nM) stained with AmyTracker. Bar, 2µm.

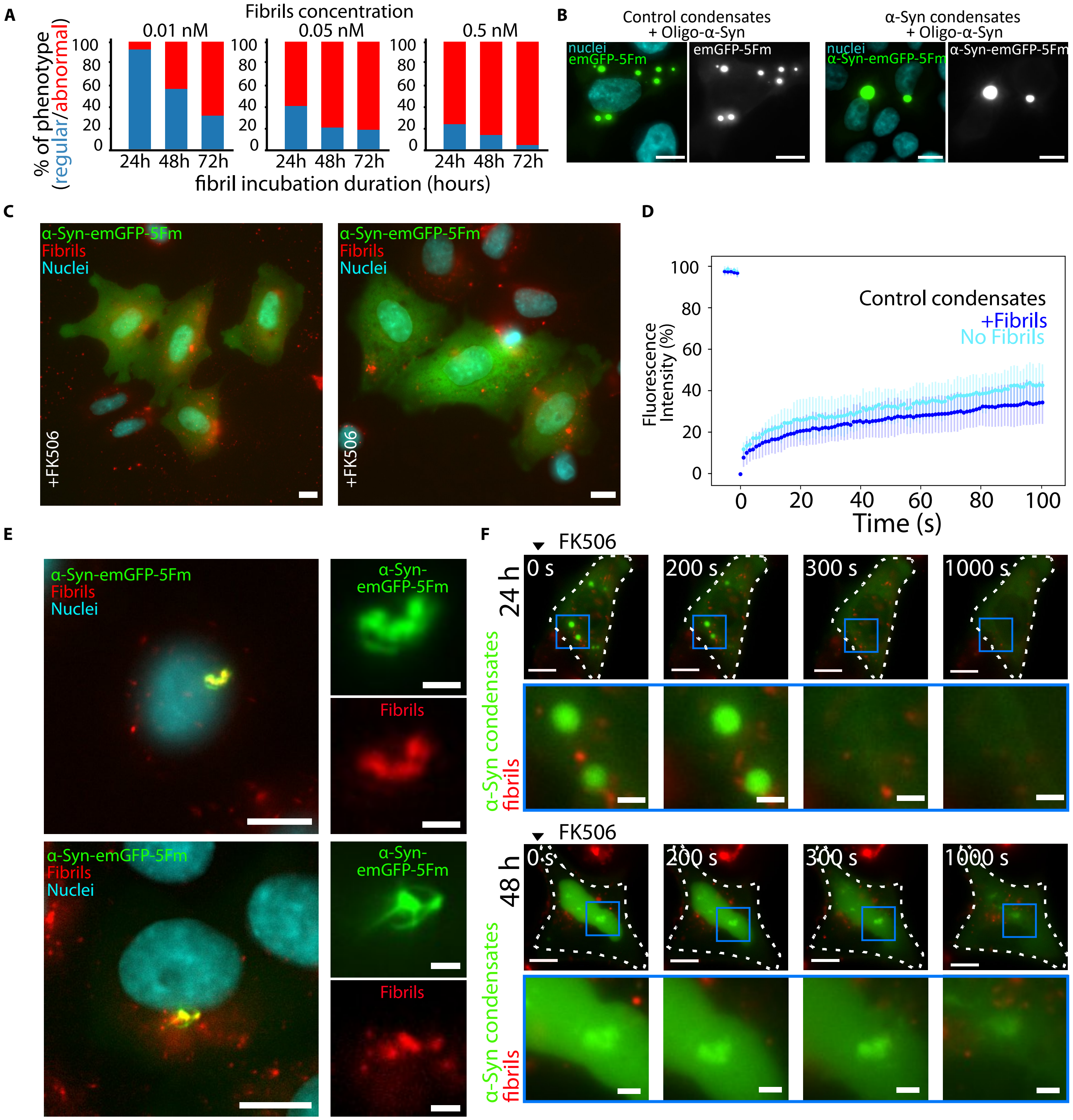

Fig. S3. Abnormally-shaped condensates occur only from α-Syn condensates exposed to fibrils (**A**)Quantification of the percentage of cells with condensates expressing the two different phenotypes: "Regular"– blue; "Abnormal" – red. as a function of time and concentration of preformed fibrils. HeLa cells expressing α-Syn-emGFP-5Fm (for 24 hours, 48 hours and 72 hours) were exposed to increasing concentration of preformed fibrils (0.01 nM, 0.1 nM and 0.5 nM). The graph is the sum of N=2 experiments. (**B**) HeLa cells expressing control (left) and α-Syn (right) condensates and exposed for 72 hours to preformed α-Syn oligomers (0.5 nM). (**C**) HeLa cells expressing α-Syn-emGFP-5Fm scaffold (green) and exposed to preformed α-Syn fibrils (red) for 72 hours in presence of FK506. Nuclei (cyan) were stained with Hoechst. Bars, 10 µm (**D**) FRAP analysis of control condensates from cells exposed (dark blue, 8 cells) or not (light blue, 15 cells) to fibrils. Pre-bleaching fluorescence intensity was set at 100 and the post-bleach fluorescence was normalized at 0 for all experiments. An acquisition was made every second. Error bars correspond to the standard deviation. (**E**) HeLa cells expressing α-Syn condensates (green) and exposed to preformed α-Syn fibrils (red) for 72 hours after incubation with FK506 for 1 hour. Nuclei (cyan) were stained with Hoechst. Bars, 10 µm for full-cell images and 2 µm for zoom-ins. (**F**) Live confocal microscopy frames depicting the FK506-driven dissolution patterns of 24 hours-old (upper pannel) and 48 hours-old (lower pannel) α-syn condensates (green) from HeLa cells exposed to fibrils (red). Different timepoints are shown, with 0 s being pinpointed at the moment when FK506 was added. Bars, 10 µm for whole cell frames and 2 µm for zoom-ins.

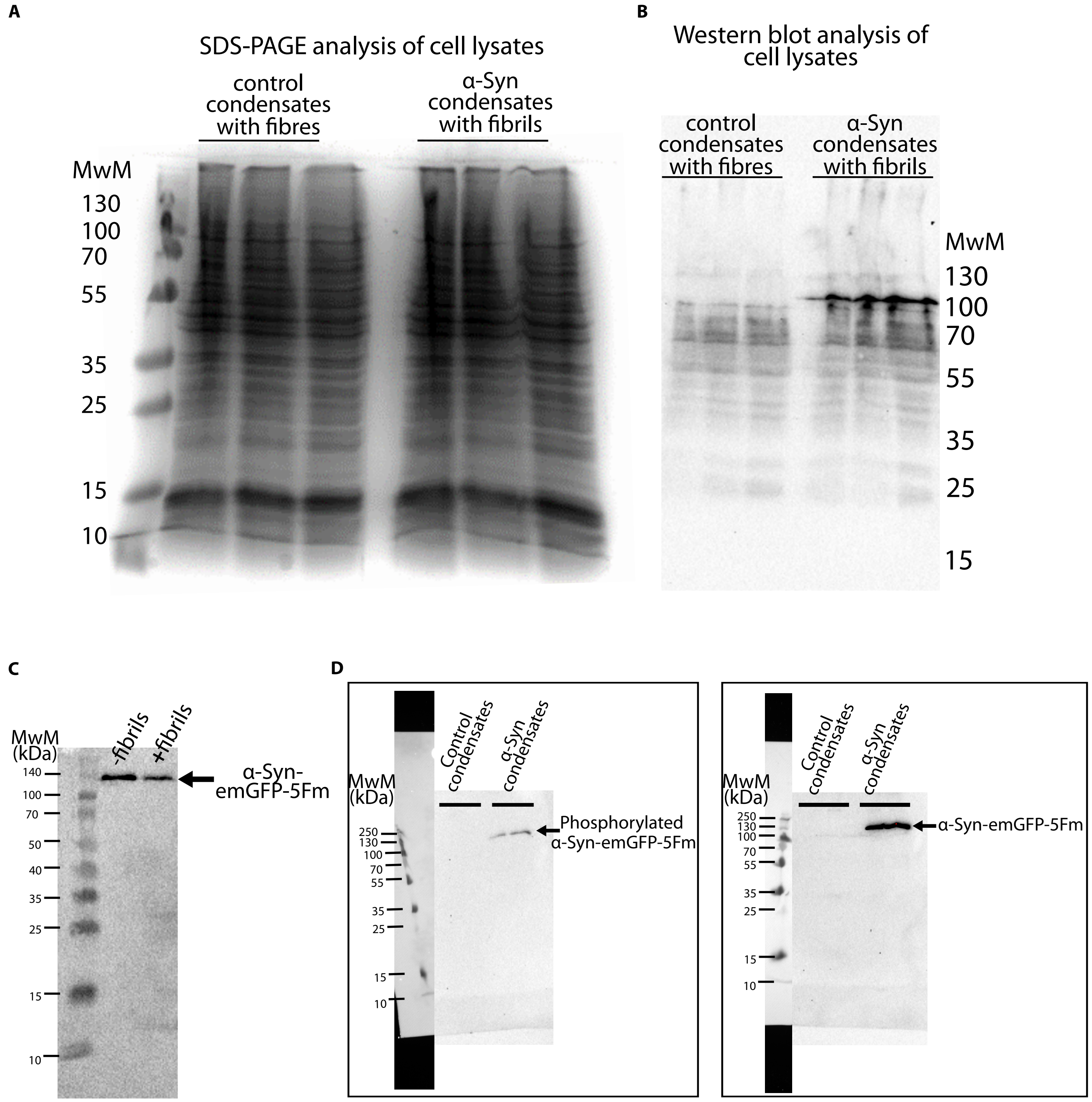

Fig. S4. Biochemical analysis of condensates with and without fibrils

(**A**) SDS-PAGE analysis of lysates used for western blot and filter trap assay. First three lanes to the left: cells expressing control condensates. Next three lanes: cells expressing α-Syn condensates. (**B**) Western Blot performed with an anti α-Syn-PS129 antibody (EP1536Y) on lysates from cells expressing control condensates (first three lanes to the left) and α-Syn condensates (fourth to sixth lanes). (**C**) Western blot performed with a anti-α-Syn primary antibody (BD Biosciences 610787) illustating the α-Syn-emGFP-5Fm construct from lysates of cells exposed or not to fibrils. (**D**) Western Blots performed with anti α-Syn (BD Biosciences 610787, MABN633 Sigma, MABN389 Sigma, # MA1-90346 Invitrogen) and an anti α-Syn-PS129 (EP1536Y) antibody on lysates from cells expressing control condensates and α-Syn condensates in presence of fibrils.

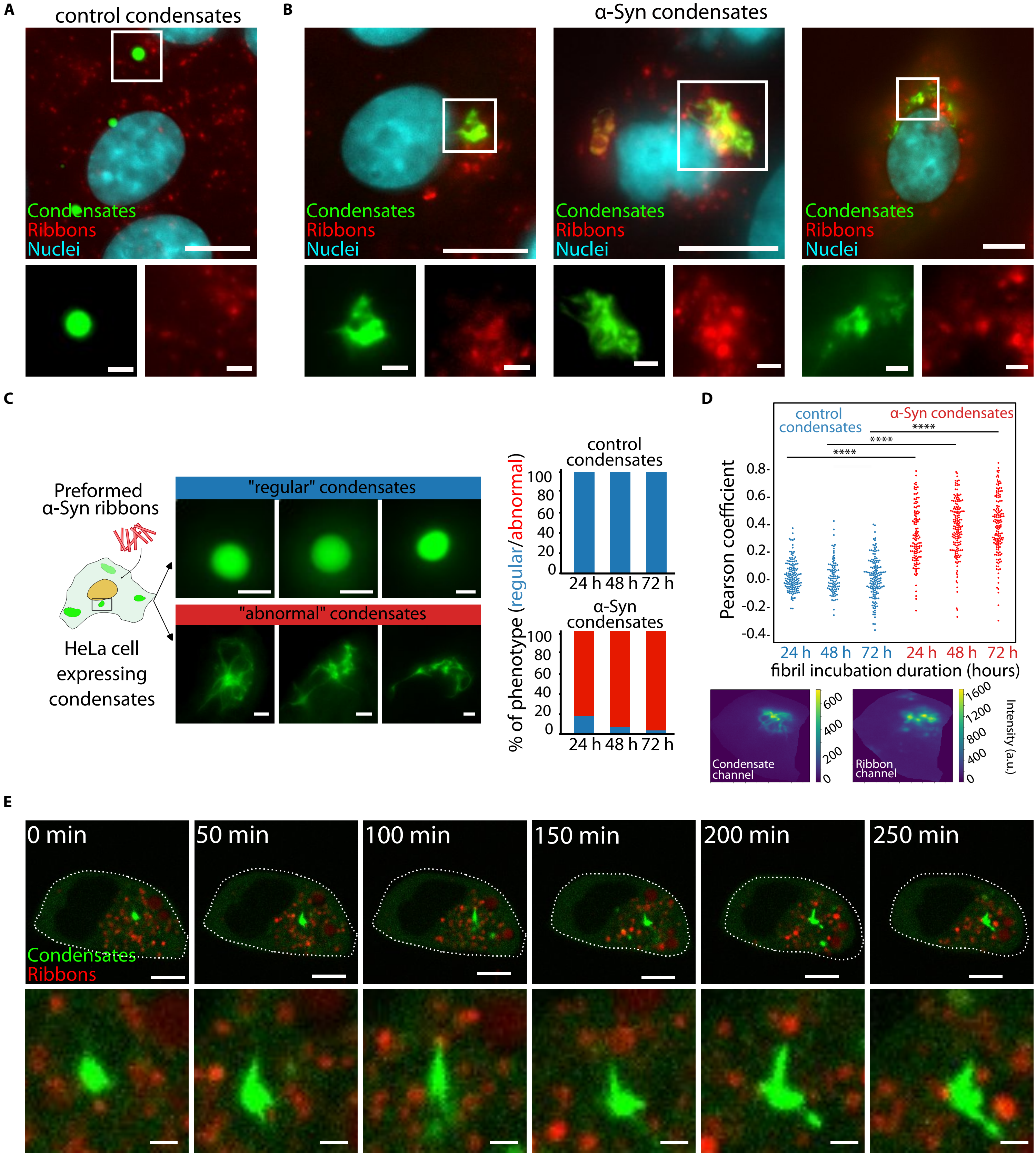

Fig. S5. α-Syn condensates are perturbed by preformed ribbons

Representative epifluorescence images of HeLa cells exposed to preformed α-Syn ribbons (red) and expressing control (**A**) and α-Syn (**B**) condensates (green). Nuclei (cyan) were stained with Hoechst. Bars, 10 µm for full-cell images and 2 µm for zoom-ins. (**C**) Quantification of the percentage of cells with condensates expressing the two different phenotypes: "regular"– blue; "abnormal" – red. The graph is the sum of N=3 experiments. (**D**) Quantification of the Pearson coefficient calculated for the correlation between the fluorescence intensities of the ribbon and condensate channels in microscopy images that were cropped to match the shape of the cell. Each point represents a cell. The graph is the sum of N=3 experiments. Differences between conditions with control and α-Syn condensates were statistically significant (p-values < 10-7 using a Student's t-test). a.u. stands for arbitrary unit. (**E**) Live confocal microscopy frames depicting HeLa cells expressing α-Syn condensates (green) changing morphology upon exposure to ribbons (red). Bars, 10 µm for full-cell images and 2 µm for zoom-ins.

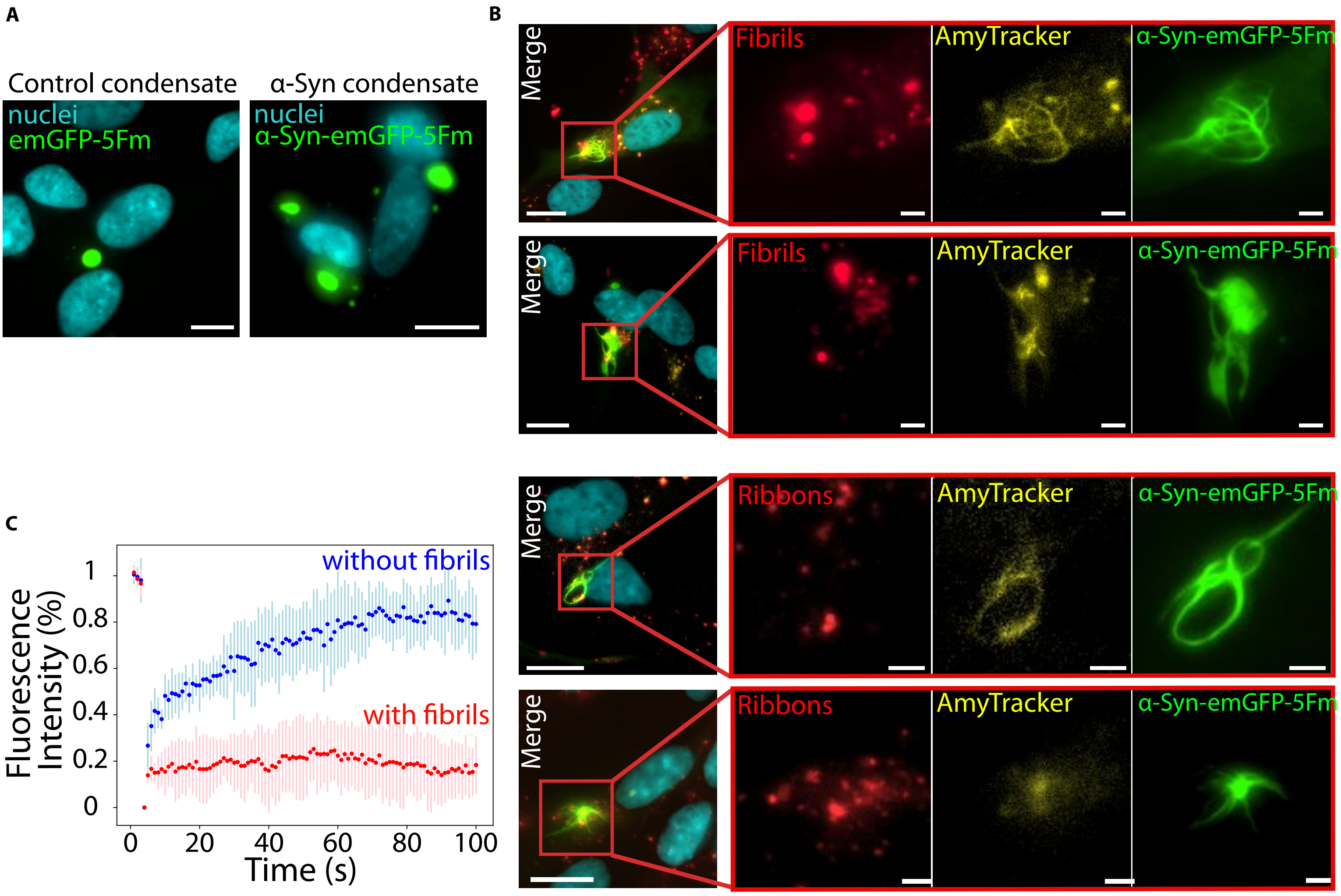

Fig. S6. Preformed α-Syn fibrils triggered the evolution of liquid α-Syn condensates into solid-needle like structures in SH-SY5Y neuronal cells.

Representative epifluorescence images of SHSY-5Y cells infected with preformed α-Syn fibrils (red) and expressing control **(A)** and α-Syn **(B)** condensates (green). Nuclei (cyan) were stained with Hoechst. AmyTracker staining on SHSY-5Y cells expressing α-Syn condensates after 72 hours incubation with fibres. Bars, 10 µm for full-cell images and 2 µm for zoom-ins. **(C)** FRAP analysis of abnormally-shaped condensates. The graph comprises the average of 10 different experiments for the condition with fibrils and 7 different experiments for the condition without fibirls . Pre-bleaching fluorescence intensity was set at 100% and the post-bleach fluorescence was normalized at 0 for all experiments. An acquisition was made every second. Error bars correspond to the standard deviation.

Construct	Oligo Name	Sequence
pcDNA3.1 α-Syn-emGFP-5Fm	LP029 HindIII_KozaK+ATG_α-SynWT	actgacaAGCTTgccaccATGGATGTATTCATGAAAGGACTTTCAAAGG
pcDNA3.1 α-Syn-emGFP-5Fm	LP030 3' SacII_α-Syn	TCAGTCCGCGGGGCTTCAGGTTCGTAGTCTTGATACC
pcDNA3.1 α-Syn(S129A)-emGFP-5Fm	ACC031 Kan Fw	CGAGCGAGCACGTACTCGGA
pcDNA3.1 α-Syn(S129A)-emGFP-5Fm	ACC032 Kan Rv	TCCGAGTACGTGCTCGCTCG
pcDNA3.1 α-Syn(S129A)-emGFP-5Fm	LP036 α-Syn for	TATGAAATGCCTGCTGAGGAAGGGTATCA
pcDNA3.1 α-Syn(S129A)-emGFP-5Fm	LP037 α-Syn Rev	TGATACCCTTCCTCAGCAGGCATTTCATA

Supplementary table 1.
List of DNA primers used for the cloning of the pcDNA3.1 α-Syn-emGFP-5Fm and pcDNA3.1 α-Syn(S129A)-emGFP-5Fm

Supplementary movies

Movie S1: Time-lapse confocal microscopy of the formation of α-Syn condensates. Acquisitions were taken every 5 minutes.

Movie S2: Time-lapse confocal microscopy of the formation of control condensates. Acquisitions were taken every 5 minutes.

Movie S3: Time-lapse epifluorescence microscopy of the dissolution of 24H-old α-Syn condensates. FK506 was added 30 seconds in. Acquisitions were taken every 10 seconds.

Movie S4: Time-lapse epifluorescence microscopy of the dissolution of 72H-old α-Syn condensates. FK506 was added 30 seconds in. Acquisitions were taken every 10 seconds.

Movie S5: Time-lapse confocal microscopy of the evolution of α-Syn condensates (green) from "regular" to "abnormal" phenotype in presence of fibres (red). Acquisitions were started 48 hours after cell exposure to fibrils and images were taken every 5 minutes.

Movie S6: Time-lapse epifluorescence microscopy of the dissolution of 72H-old α-Syn condensates (green) with fibres (red). FK506 was added 30 seconds in. Acquisitions were taken every 10 seconds.

Movie S7: Time-lapse confocal microscopy of the growth of α-Syn condensates (green) in presence of fibres (red). Acquisitions were taken every 5 minutes.

Movie S8: Time-lapse confocal microscopy of the evolution of α-Syn condensates (green) from "regular" to "abnormal" phenotype in presence of ribbons (red). Acquisitions were started 48 hours after cell exposure to ribbons and images were taken every 5 minutes
